# Research Priority Setting Exercises in Neglected Tropical Diseases: A Systematic Mapping Review

**DOI:** 10.1007/s44197-026-00614-y

**Published:** 2026-07-11

**Authors:** Manish Barik, Sushree Nibedita Panda, Gooty Shirisha, Prabhu Kalyan Das, Bhaskar Tiwary

**Affiliations:** 1 New Delhi, India; 2 The George Institute for Global Health, Hyderabad, India; 3 School of Medical Sciences, University of Hyderabad, Hyderabad, India; 4 ICMR Regional Medical Research Center, Bhubaneswar, Odisha India; 5 Centre for Research in Schemes and Policies Hyderabad, Hyderabad, Telangana India; 6 Manipal Academy of Higher Education, Manipal, Karnataka India

**Keywords:** Neglected tropical diseases, Research priority-setting, Systematic review, Stakeholder engagement, Health policy, Equity

## Abstract

**Background:**

Neglected tropical diseases (NTDs) affect more than 1.7 billion people worldwide, primarily in low- and middle-income countries. Despite their high burden, NTDs remain underfunded and receive fragmented research attention. Research priority-setting provides a systematic approach to align limited resources with the most pressing needs. While several priority setting exercises have been conducted for NTDs, their findings have not been synthesized. This study aimed to systematically map the published research priority-setting exercises for NTDs, synthesize their methodologies, stakeholder involvement, and thematic priorities.

**Methodology:**

We conducted a systematic mapping review of studies published in PubMed, EMBASE, and Web of Science up to March 28, 2025. Eligible studies were those that explicitly reported research prioritization for at least one WHO-listed NTD. Data were extracted on study design, geographic scope, prioritization methods, stakeholder engagement, and outcomes, and were analyzed descriptively. Reported priorities were classified into six domains: (1) health systems and service delivery, (2) diagnostics and therapeutics, (3) surveillance and data systems, (4) community engagement and health education, (5) integration and coordination, and (6) equity, policy, and implementation research.

**Results:**

Of the 1,678 records, 15 studies met inclusion criteria. Most were mixed-methods (*n* = 10) and conducted at the country (*n* = 5) or regional level (*n* = 5). Delphi was the most used framework (*n* = 6). Fourteen (out of 15) studies engaged clinicians, policymakers, and researchers; 12 involved NGOs; 8 included vulnerable populations; 6 included patients. Lymphatic filariasis was the most frequently prioritized NTD (*n* = 7), followed by soil-transmitted helminths and schistosomiasis (*n* = 5 each). Research priorities clustered around health system strengthening (6/15 studies), community engagement (7/15 studies), and equity-oriented implementation research (10/15 studies).

**Conclusion:**

Research priority-setting for NTDs has expanded in recent years but remains heterogeneous in methodology, stakeholder inclusiveness, and transparency. Findings also call for caution against disease prioritization approaches that focus only on high-prevalence NTDs which risk overlooking less common NTDs and the populations affected by them. Six cross-cutting domains of research priorities were identified across the studies, largely consistent with the WHO NTD Roadmap. The identified domains can guide WHO regional offices and national programs in updating research roadmaps, explicitly linking priorities to policy and implementation pathways to accelerate progress toward global NTD targets.

**Supplementary Information:**

The online version contains supplementary material available at https://doi.org/10.1007/s44197-026-00614-y.

## Introduction

Neglected Tropical Diseases (NTDs) are a diverse group of poverty-associated infections caused by viruses, bacteria, parasites, fungi or toxins [1]. NTDs disproportionately affect more than 1.7 billion people annually, primarily in low- and middle-income countries across tropical and sub-tropical regions [1]. Unlike many infectious diseases, NTDs rarely cause large-scale mortality but are strongly associated with chronic disability and stigma that reduce quality of life and productivity [2, 3]. The Global Burden of Disease (GBD) 2019 study estimated that NTDs accounted for over 18 million disability-adjusted life years (DALYs), with conditions such as lymphatic filariasis, leprosy, onchocerciasis, and snakebite envenoming causing long-term impairments and social exclusion. Skin-related NTDs, in particular, contribute to visible disabilities and social discrimination, reinforcing cycles of poverty [4, 5]. Despite this global impact, NTDs historically attracted only a fraction of the global health research funding [6].

The co-endemic nature of NTDs, combined with complex transmission dynamics, further complicates control efforts and highlights the need for coordinated and integrated research agendas [7]. Analyses of the recent GBD assessments have highlighted both high disability-adjusted life years attributable to NTDs and the fragmented coordination of control programs, which are often siloed by disease or region [8, 9]. This fragmentation hampers the development of integrated strategies and leaves key knowledge gaps unaddressed, particularly in diagnostics, therapeutics, and health systems research and strengthening [10, 11]. The WHO’s NTD Roadmap 2021–2030 now sets ambitious targets – including a 90% reduction in people requiring treatment and a 75% reduction in NTD-related DALYs by 2030 underscoring the urgent need for intensified research and programmatic action to achieve these goals [12]. These highlight the essential role of NTD control in achieving health equity and the broader Sustainable Development Goals (SDG) agenda.

Recent WHO report on NTD shows a steep 41% decline in official development assistance (ODA) for NTDs between 2018 and 2023, falling from about US$ 440 million to US$ 260 million. Most of this funding came from three donors – the United States, the United Kingdom, and the Bill & Melinda Gates Foundation [13]. Also, there is no pooled financing mechanism for NTDs, unlike malaria or HIV programs. This funding gap makes sustained research and implementation uncertain, especially in low-income countries. The report also warns that such shortfalls threaten elimination progress and calls for stronger national ownership, domestic resource mobilization, and integration of NTD work into primary health care [13].

Health research priority-setting (RPS) is the process of deciding which health research questions or areas should be prioritized. This includes what many organizations describe as “priority-setting exercises,” “strategic planning,” or “agenda setting.” By engaging diverse stakeholders ranging from affected communities and frontline health workers to policymakers and funders, RPS can enhance transparency, equity, and impact [14, 15]. These processes aim to balance scientific feasibility with public health relevance, thereby enhancing transparency and strengthening accountability in decision-making. In global health, RPS has been applied across diverse domains [16–18].

Priority-setting in NTDs is conceptually distinct from other health research fields. Unlike diseases managed through single-pathogen programs, NTDs share ecological overlap, co-endemic distribution, and strong links to poverty and social exclusion. Thus, the priority setting in NTDs must integrate multisectoral inputs spanning health, sanitation, and environment while maintaining a focus on equity and community participation. Several individual priority setting exercises have been conducted for NTDs -at global, national or disease-specific levels, but their findings have not been synthesized [19].This lack of synthesis constrains the ability of researchers, funders, and policymakers to identify consensus priorities, benchmark progress, or assess alignment with global strategies.

This systematic mapping review addresses these gaps by collating and critically appraising existing RPS exercises for NTDs. It aims to synthesize their objectives, methodological approaches, stakeholder involvement, and reported priorities. By doing so, the review seeks to highlight areas of convergence, reveal underexplored research domains, and provide evidence to guide future NTD research agendas in line with global disease burden and elimination targets.

## Methods

This systematic mapping review was prospectively registered with PROSPERO (CRD420251021734.) and adopted the PRISMA 2020 guideline [20]. All procedures followed the pre-registered protocol to ensure consistency in the study’s design, methodology, and analysis .

### Search Strategy

We performed a comprehensive search in PubMed, EMBASE and Web of Science, covering all records from database inception through 28 March, 2025. Our strategy combined each platform-specific controlled vocabulary with relevant keywords specifically *“research priority” AND “NTDs” or “neglected tropical diseases.”* All retrieved citations were exported and organized using Zotero and deduplicated. All retrieved records were then screened against predefined inclusion and exclusion criteria. The detailed search strategy is given in the Supplementary file.

### Inclusion and Exclusion Criteria

This review included studies that applied a structured process to identify, rank, score, or reach consensus on research priorities addressing at least one WHO-listed NTD [1]. To be eligible, studies had to clearly report the the objective of the prioritization exercise. We imposed no date restrictions but excluded publications not written in English. We excluded non-empirical studies (such as commentaries, editorials, discussion papers, and opinions), secondary studies, guidelines, books, and book chapters.

### Study Screening and Selection

Four authors (MB, SNP, PKD and GS) independently assessed the titles and abstracts in the first phase followed by full-text screening of potentially relevant studies performed by SNP, PKD, & GS using Rayyan platform. Each article was double screened (in both the phases) to maintain the quality of the review. Disagreements or conflicts that arose during the screening process were resolved through online discussions with the third author (MB) to reach a final consensus.

### Data Extraction

We developed a preliminary data extraction form referring to the ten domains in REporting guideline for PRIority SEtting of health research (REPRISE) [21]. We piloted and modified the data extraction form with two included studies. For each document, we extracted its basic characteristics such as publication year, journal or report source, country or setting, geographical focus, targeted NTD(s), type of prioritization exercise, funding sources, and conflict of interest statements, before detailing its methodological process. This included the composition of the leadership team, the specific framework or method employed, the sequential stages of prioritization, and the year the exercise was conducted (defaulting to the publication year if unspecified). We also recorded stakeholder involvement, including stakeholder types and engagement methods (online or in-person); as well as techniques used to identify and compile research priorities. Finally, we captured the outcomes by listing the priority items in each study. All team members (SNP, PKD and GS) participated in the data extraction. Each article was double extracted by two reviewers. Any discrepancies that arose were resolved via discussion with a third author (MB). The included studies describe research priority setting processes and methodologies rather than outcomes or effectiveness. We did not conduct a formal quality appraisal because reporting varied widely across studies. Many studies lacked sufficient methodological detail to support consistent scoring across appraisal criteria. This review therefore focused on mapping methodological approaches, stakeholder engagement, and reported priority themes in NTD research priority setting rather than judging overall study quality.

### Data Analysis and Synthesis

We used Microsoft Excel to perform descriptive analysis. Visualizations were developed in Google Looker Studio to present patterns of key characteristics, including year of publication, geographic focus, study design, stakeholder groups, and funding sources. These were summarized using frequencies and proportions to identify patterns and descriptive trends. We assessed the methodological approaches used for research priority setting by summarizing the types of prioritization methods employed, and the modes of stakeholder engagement (e.g., online, in-person, or both). The composition of stakeholder groups involved in each study was also analyzed, capturing the types and diversity of participants, including clinicians, researchers, policymakers, donors, patients, and representatives of vulnerable populations. Additionally, we examined the thematic focus of the included studies by calculating the number and proportion of studies addressing specific NTDs, and distinguishing between those that focused on skin-related versus non-skin-related NTDs.

The research team reviewed the WHO NTD Roadmap 2021–2030 and its strategic pillars, along with related WHO research strategies and monitoring frameworks, to identify broad thematic areas that encompass major dimensions of NTD control and research [12]. The six predefined domains were (1) health systems and service delivery, (2) diagnostics and therapeutics, (3) surveillance and data systems, (4) community engagement and health education, (5) integration and coordination(across diseases and sectors*)*, and (6) equity, policy, and implementation research.

Each extracted priority was mapped to the most relevant domain based on its primary focus. When a priority overlapped with more than one area, classification was guided by consensus among the authors. This approach enabled aggregation of diverse and context-specific priorities into structured domains that are directly linked to the global agenda for NTD research and action.

## Results

**Fig. 1:**
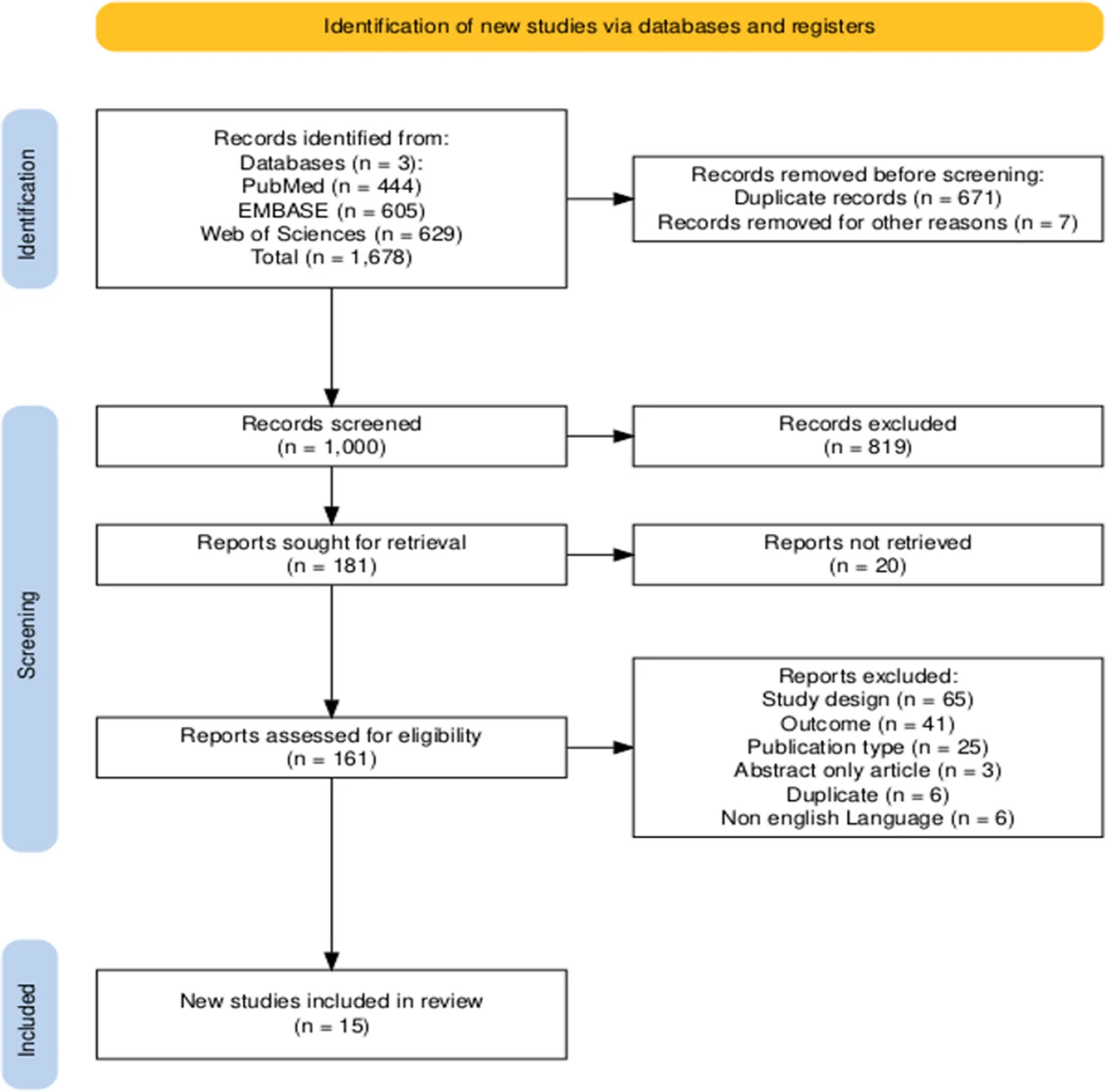
PRISMA flow diagram of study selection for the review

The initial database search found 1,678 records from PubMed (*n* = 444), Embase (*n* = 605), and Web of Science (*n* = 629). After removing 671 duplicates and seven records for other reasons(books etc.), 1,000 unique records were screened by title and abstract. Of these, 819 were excluded, leaving 181 full-text articles sought for retrieval. Twenty reports could not be retrieved, and 161 full-text articles were assessed for eligibility. A total of 146 articles were excluded for not fulfilling eligibility criteria. Ultimately, 15 studies met the inclusion criteria and were included in the final synthesis [22–36]. A detailed PRISMA flow diagram is presented in Fig. 1.

### Journals and Geographic Distribution of Corresponding Authors

**Fig. 2:**
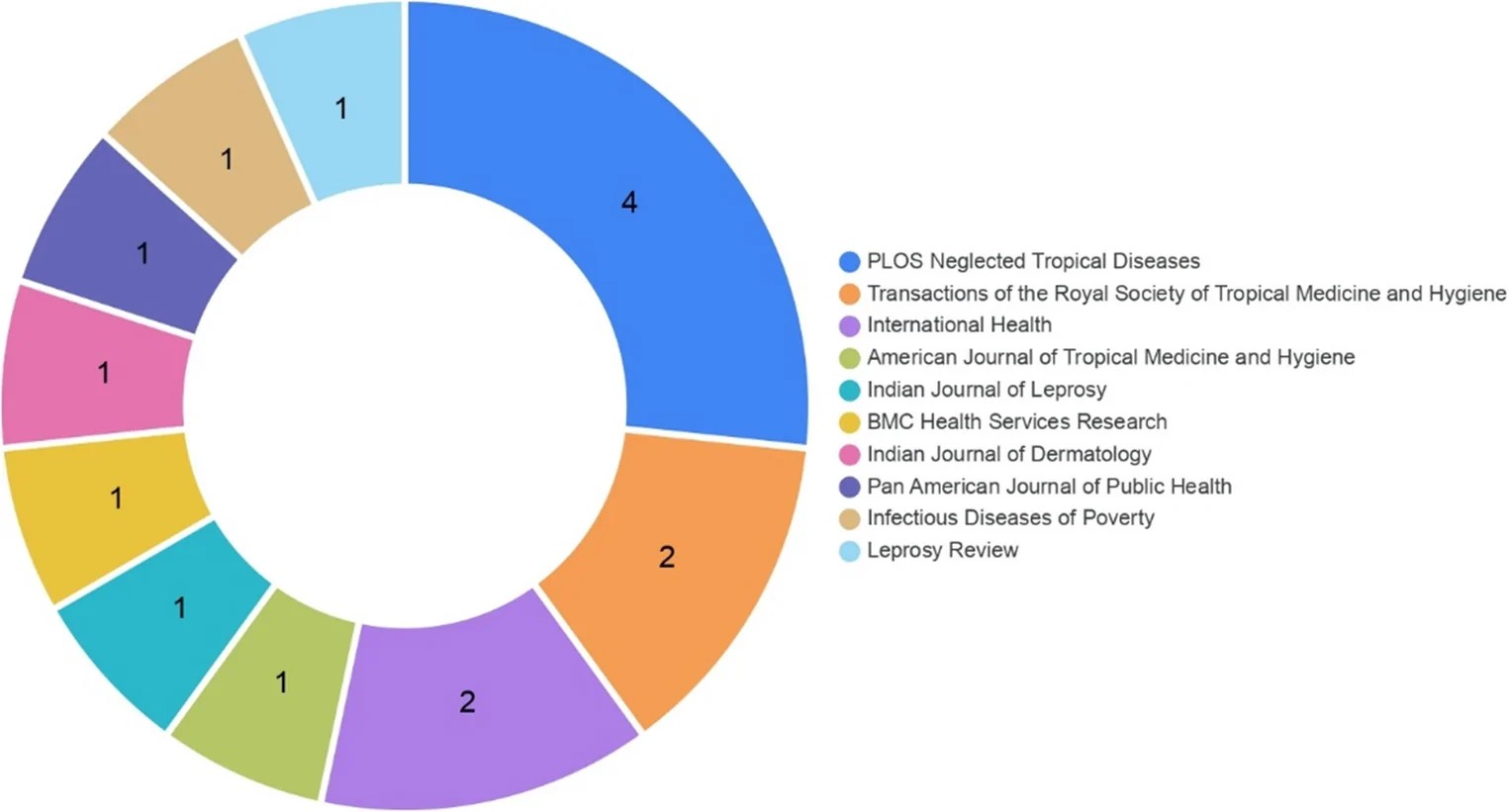
Journals publishing NTD research priority-setting studies

This review included 15 studies published across diverse peer-reviewed journals, with *PLOS Neglected Tropical Diseases* being the most frequent publication outlet (*n* = 4). Journals ranged from international outlets like *International Health*, *Transactions of the Royal Society of Tropical Medicine and Hygiene*, and *American Journal of Tropical Medicine and Hygiene*, to regional journals such as the *Indian Journal of Leprosy* and *Leprosy Review in* Fig. 2. Most of the corresponding authors were affiliated with institutions based in high-income countries (10/15 studies), including the United States of America (2 studies), United Kingdom (4 studies), The Netherlands (3 studies), and Canada (one study) (Table 1).

**Table 1:** Characteristics of included priority-setting studies

Study ID	Title of the Paper	Journal	Affiliation of corresponding author	Objective of the study	Study Design	Geographic focus of the study	Funding status
Walsh 2018, [22]	A Delphi consultation to assess indicators of readiness to provide quality health facility based lymphoedema management services	PLOS Neglected Tropical Diseases	United States of America	The study aimed to establish consensus on key domains and indicators to evaluate national program readiness for delivering quality health facility-based lymphoedema management services, as part of WHO’s toolkit to guide MMDP (Morbidity Management and Disability Prevention) implementation and evaluation.	Quantitative	Global	USAID, United States
Neevel 2018, [23]	A research agenda to reinforce rabies control: A qualitative and quantitative prioritization	PLOS Neglected Tropical Diseases	The Netherlands	The study aimed to assess and prioritize research needs that could enhance current strategies or lead to the development of novel approaches for rabies control, particularly by incorporating perspectives from key opinion leaders across disciplines using a structured research framework based on the epidemiological triangle.	Mixed methods	Regional	Not funded
Waite 2017,[24]	Collaborating to develop joint water, sanitation and hygiene (WASH) and neglected tropical disease (NTD) sector monitoring: an expert consultation	International Health	United Kingdom	The study aimed to identify a core set of measurable priority indicators for joint monitoring between the WASH and NTD sectors, through expert consultation using a formal Delphi method, in order to enhance sector collaboration and guide integrated program monitoring from the perspective of NTDs.	Qualitative	Global	Not funded
Aglanu 2023, [25]	Community members and healthcare workers’ priorities for the control and prevention of snakebite envenoming in Ghana.	PLOS Neglected Tropical Diseases	The Netherlands	The study aimed to identify the research and management priorities related to snakebite envenoming from the perspectives of community members and healthcare workers in two high-prevalence regions in Ghana, and compare them with WHO’s strategic objectives to guide locally responsive policy and research agendas.	Qualitative	Country specific	Royal Society of Tropical Medicine and Hygiene (UK), Hamish Ogston Foundation (UK), Buruli Ulcer Foundation Groningen (The Netherlands)
Baghotia 2021, [26]	Delphi Study on Eliminating Leprosy in India	Indian Journal of Leprosy	India	The study aimed to identify the major challenges and implementation gaps in the National Leprosy Eradication Program (NLEP) in India through a Delphi process involving experts, with the goal of informing future actions to achieve zero leprosy incidence.	Mixed methods	Country specific	Unclear
Dagenais 2018, [27]	Establishing research priorities in prevention and control of vector-borne diseases in urban areas: a collaborative process	Infectious Diseases of Poverty	Canada	The study aimed to collaboratively identify research priorities in the prevention and control of vector-borne and other infectious diseases of poverty in urban areas by conducting a concept mapping exercise involving international researchers and decision-makers, based on six preceding scoping reviews and expert consultations.	Mixed methods	Regional	WHO/Tropical Disease Research, Switzerland
Ng’etich 2021, [28]	Feasibility of implementing recommendations to improve neglected tropical diseases surveillance and response in Kenya: a modified Delphi study	BMC Health Services Research	South Africa	The study aimed to assess the feasibility of implementing recommendations to improve surveillance and response activities for preventive chemotherapy neglected tropical diseases (PC-NTDs) in Kenya, based on the perspectives of health stakeholders at the sub-national level, using a structured Delphi consensus process.	Mixed methods	Country specific	WHO /Tropical Disease Research, Switzerland
Narahari 2017, [29]	Future Research Priorities for Morbidity Control of Lymphedema	Indian Journal of Dermatology	India	This paper summarizes the process of priority setting partnership (PSP) in lymphedema, discussions during the final prioritization workshop (FPW), and recommendation on the top seven priorities for future research in lymphedema and a brief roadmap.	Mixed methods	Not Specified	Indian Council of Medical Research(ICMR), Govt of India
Deribe 2015, [30]	Measuring elimination of podoconiosis, endemicity classifications, case definition and targets: an international Delphi exercise	International Health	United Kingdom	The study aimed to produce standardized case definitions, endemicity classifications, elimination targets, and clinical outcome indicators for podoconiosis, using expert consensus, to support disease monitoring, evaluation, and elimination efforts.	Qualitative	Regional	Wellcome Trust, United Kingdom (UK)
Simpson 2021, [31]	Research priorities for control of zoonoses in South Africa	Transactions of the Royal Society of Tropical Medicine and Hygiene	South Africa	The study aimed to identify research priorities for the control of zoonotic diseases in South Africa by applying a systematic, expert-driven framework that evaluates and scores different types of research, with the goal of informing more effective and integrated prevention and control strategies across animal and human health sectors.	Mixed methods	Country specific	Belgian Directorate General for Development Cooperation and Humanitarian Aid, Belgium
Simpson 2023, [36]	Research priorities to support the development of integrated national strategies to control skin-neglected tropical diseases	Transactions of the Royal Society of Tropical Medicine and Hygiene	United Kingdom	The study aimed to identify and summarize operational research priorities to support the development and implementation of national integrated strategies for controlling skin-neglected tropical diseases (skin-NTDs), with a focus on informing country-level strategies through evidence-based recommendations.	Qualitative	Regional	Bill & Melinda Gates Foundation (USA), UK Aid (UK), and USAID (USA)
Khazai 2019, [32]	Reviewing Research Priorities of the Leprosy Research Initiative (LRI): a stakeholder consultation	Leprosy Review	The Netherlands	The Leprosy Research Initiative (LRI) provides competitive research funding for research projects related to one of five research priorities. Five years after its launch in 2013 and with new developments in the field, the LRI consulted a wide range of stakeholders to evaluate its research priorities and to contribute to a wider research agenda	Mixed methods	Global	Unclear
Cediel 2013, [33]	Setting priorities for surveillance, prevention, and control of zoonoses in Bogota, Colombia	Pan American Journal of Public Health	Italy	The study aimed to establish a list of priority zoonotic diseases in Bogota, Colombia, through a participatory process involving experts and key stakeholders, in order to guide public health actions related to the surveillance, prevention, and control of zoonoses.	Mixed methods	Country specific	Not funded
Delea 2024, [34]	Slaying the Serpent: A Research Agenda to Expand Intervention Development and Accelerate Guinea Worm Eradication Efforts	American Journal of Tropical Medicine and Hygiene	United States of America	The study aimed to develop and expand a research agenda for the global Guinea Worm Eradication Program (GWEP) that addresses evidence gaps and eradication endgame challenges by using a systems-informed One Health approach. It also sought to prioritize and guide research efforts that could enhance surveillance, diagnostics, therapeutics, and intervention strategies to accelerate progress towards certification of Guinea worm eradication by 2030.	Mixed methods	Global	The Carter Center
Stienstra 2023, [35]	Stakeholder perspectives from 15 countries in Africa on barriers in snakebite envenoming research and the potential role of research hubs	PLOS Neglected Tropical Diseases	United Kingdom	Study engaged snakebite stakeholders from various disciplines in 15 countries in the WHO Africa region to identify barriers to snakebite evidence-based decision-making at the national and regional levels and to explore whether developing regional research hubs as a research and policy initiative could help to overcome these barriers	Mixed methods	Regional	Wellcome Trust, UK

### Study Design and Geographic Scope

**Fig. 3:**
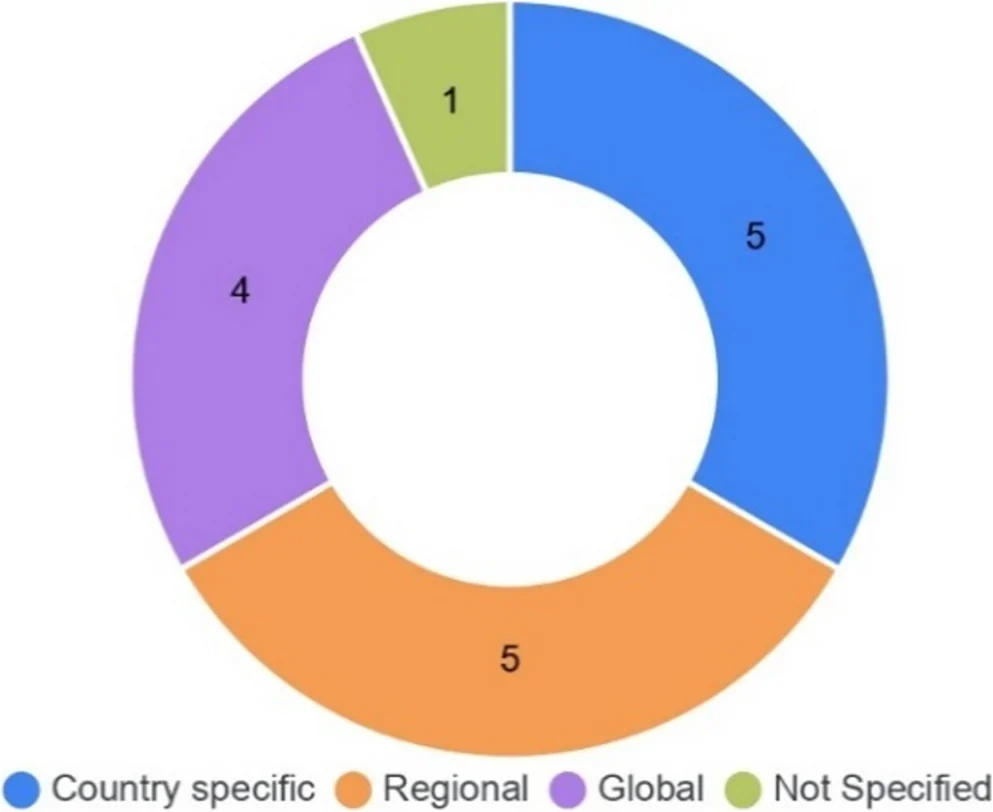
Distribution of focus areas in NTD research priority-setting studies

Among the included studies, mixed methods designs were the most frequently adopted design (*n* = 10) [23, 26–29, 31–35], followed by qualitative approaches (*n* = 4) [24, 25, 30, 36], and one study employed quantitative methods [22]. The majority of studies were conducted at country-specific (*n* = 5) or regional levels (*n* = 5), with only a few having a global focus (*n* = 4) and one study did not specify the geographical focus (Fig. 3**)**. Countries most frequently represented in the priority setting processes were India, Ghana, Kenya, Ethiopia, Colombia, and South Africa.

### Methods Used for Priority Setting

**Fig. 4:**
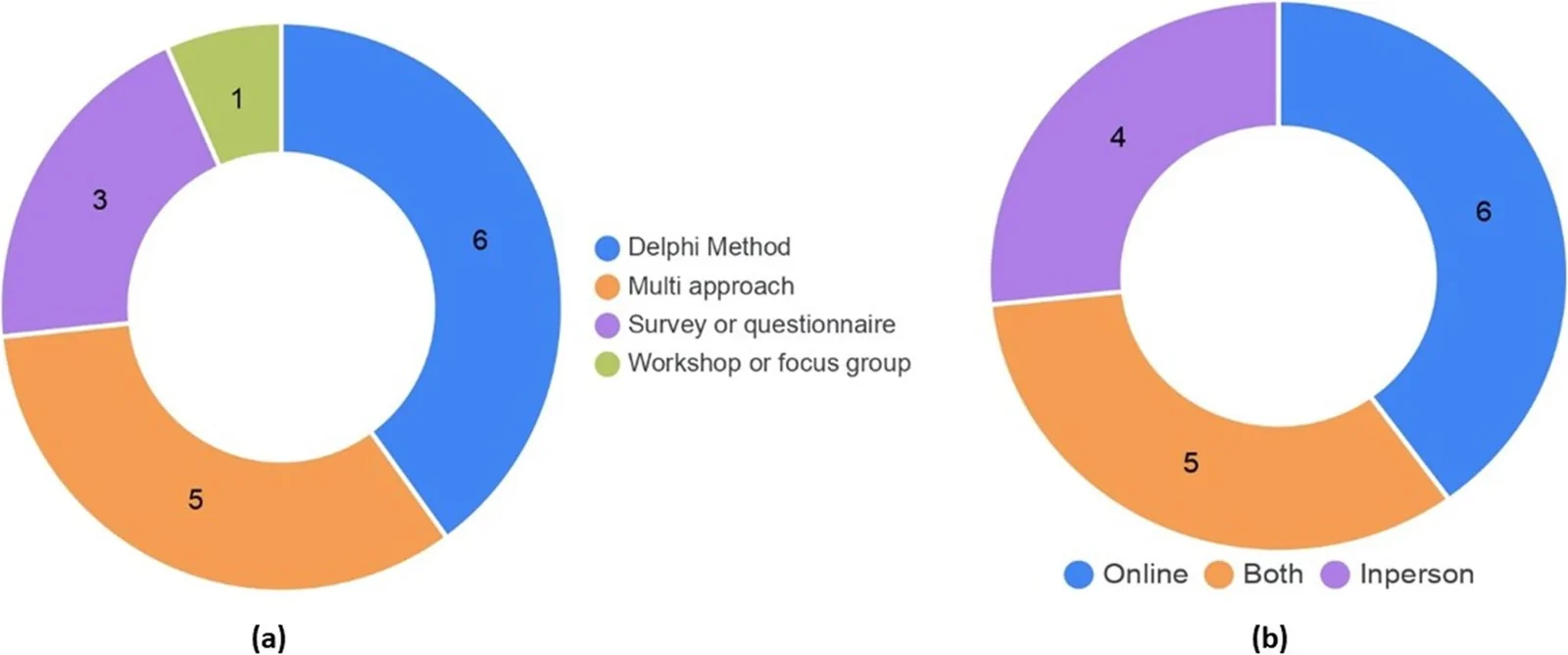
(**a**) Methods used in priority-setting and (**b**) modes of stakeholder engagement

The most common method employed in the included studies was a “Delphi” strategy (*n* = 6). A total of 5 studies used a “multi approach” which often involved combinations of surveys, interviews, workshops, or literature reviews. Frameworks such as Child Health and Nutrition Research Initiative (CHNRI) and the James Lind Alliance framework were used in one study each. Online engagement was the most common mode of data collection (*n* = 6), followed by both online and in-person methods (*n* = 5), and four studies used only in-person engagement in Fig. 4.

### Stakeholder Engagement

**Fig. 5:**
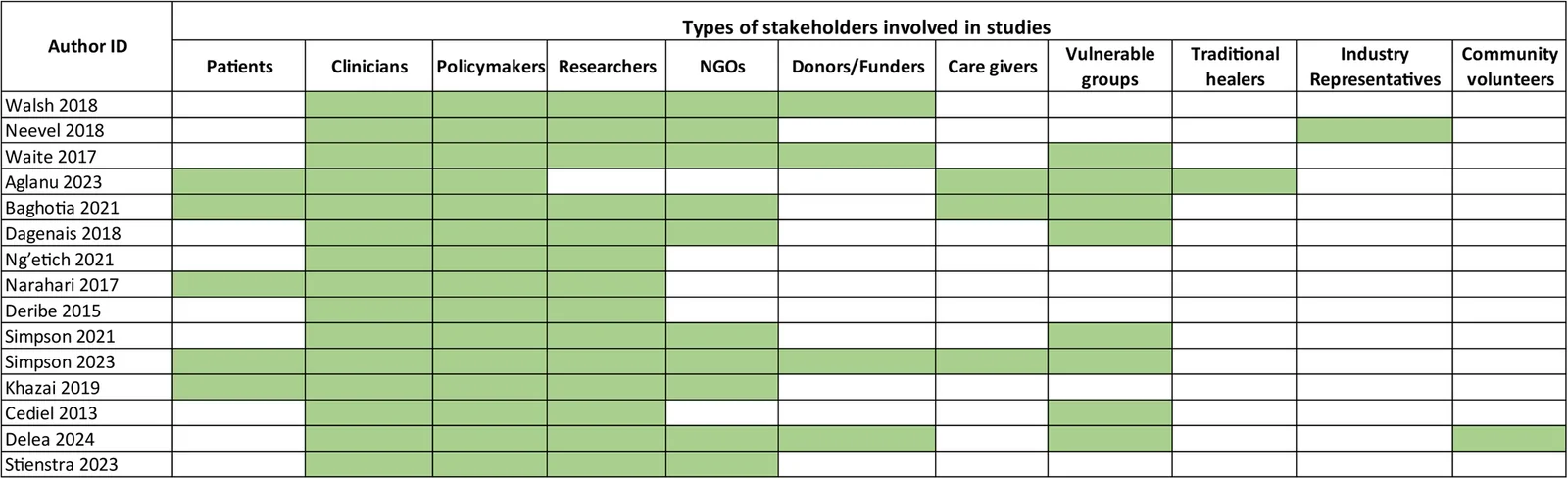
Types of stakeholder engagement across studies

All 15 studies engaged multiple stakeholder groups in their research priority-setting processes. Clinicians or healthcare providers and policymakers were included in 15 of the studies. Non-governmental organizations (NGOs) participated in 11 studies, and researchers in 14 studies, while specific groups such as vulnerable or marginalized populations were involved in 8 studies. Patients were included in 6 studies, while caregivers were represented in 3. Donors or funders participated in 4 studies. Less frequently represented groups included traditional healers (*n* = 1), community volunteers (*n* = 1) and industry representatives (*n* = 1) Fig. 5.

### Reporting of Funding and Conflict of Interest

Funding support was reported in 10 studies. Key funders included USAID, WHO/TDR, the Carter Center, the Wellcome Trust, UK Aid, the Hamish Ogston Foundation, and national government agencies(example: ICMR, India) (Table 1). Two studies did not declare their funding sources, and three explicitly reported receiving no funding. Declarations of conflict of interest were largely absent; only one study(1/15) reported having no conflicts.

### Diversity in Diseases Prioritized

Across the included studies, research priority-setting exercises covered a diverse range of neglected tropical diseases (NTDs). Lymphatic filariasis was the most frequently addressed, appearing in seven studies [22, 24, 27–30, 36]. Four studies each focused on leprosy [24, 26, 32, 36] and soil-transmitted helminths [24, 28, 33, 36]. Schistosomiasis and trachoma were included in three studies each [24, 28, 36], while leishmaniasis was highlighted in three studies [27, 33, 36]. Two studies each examined rabies [23, 31], Chagas disease [27, 33], and snakebite envenoming [25, 35]. Other NTDs such as Buruli ulcer, scabies, yaws, dengue, brucellosis and Guinea worm disease were represented in only one study each. Of the 15 included studies, 11 (73%) addressed one or more skin-related NTDs, either as a primary or secondary focus (Table 2).

**Table 2:** Number of studies and key authors by NTD focus

NTDs	No. of Studies	Key Authors
Lymphatic Filariasis	7	Walsh 2018, Waite 2017, Dagenais 2018, Ng’etich 2021, Narahari 2017, Deribe 2015, Simpson 2023
Leprosy	4	Waite 2017, Baghotia 2021, Simpson 2023, Khazai 2019
Soil Transmitted Helminths	4	Waite 2017, Ng’etich 2021, Simpson 2023, Cediel 2013
Schistosomiasis	3	Waite 2017, Ng’etich 2021, Simpson 2023
Trachoma	3	Waite 2017, Ng’etich 2021, Simpson 2023
Leishmaniasis	3	Dagenais 2018, Simpson 2023, Cediel 2013
Rabies	2	Neevel 2018, Simpson 2021
Chagas Disease	2	Dagenais 2018, Cediel 2013
Snakebite Envenoming	2	Aglanu 2023, Stienstra 2023
Buruli Ulcer, Scabies, Yaws, Dengue, Brucellosis, and Guinea Worm Disease (Dracunculiasis)	1 each	Simpson 2023, Dagenais 2018, Simpson 2021, Cediel 2013, Delea 2024

### Classifications of Research Priorities Across Thematic Domains

**Fig. 6:**
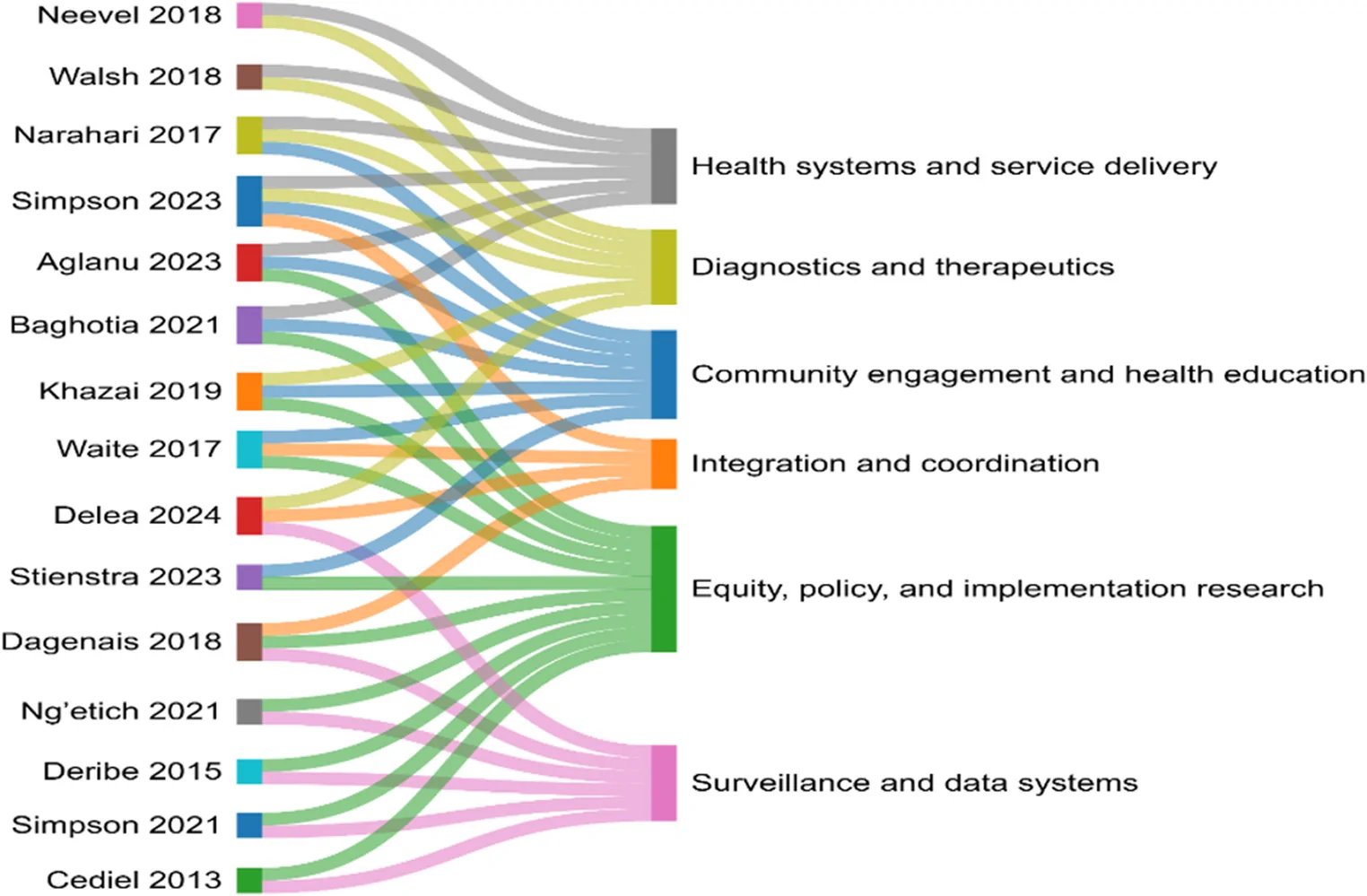
Mapping of included studies to priority domains

From the 15 included studies, a wide range of research priorities were reported, reflecting variations in methodology and stakeholder perspectives. The priorities clustered into six predefined thematic domains: (1) health systems and service delivery, (2) diagnostics and therapeutics, (3) surveillance and data systems, (4) community engagement and health education, (5) integration and coordination (across diseases and sectors*)* and (6) equity, policy, and implementation research. These domains span multiple disease areas and represent the main pillars around which research priority-setting in NTDs has been organized in Fig. 6.

1. Health systems and service delivery.
  This was the prominent domain, identified six studies (out of 15) [22, 23, 25, 26, 29, 36]. Priorities included improving clinical care, optimizing supply chains for essential medicines, and enhancing the delivery and quality of services. Walsh 2018, Aglanu 2023, Baghotia 2021 identified the urgent need for capacity building among frontline healthcare providers, particularly in managing diseases like leprosy, lymphatic filariasis, and snakebite envenoming [22, 25, 26]. This encompassed both initial and refresher training and the development of clinical guidelines for accurate diagnosis and appropriate case management.
  Moreover, health system strengthening was discussed in relation to referral mechanisms, availability of antivenoms and antibiotics, and the provision of infrastructure and equipment. For example, Aglanu 2023 highlighted the need for emergency transportation, oxygen support, and logistical resources to reduce treatment delays for snakebite victims [25]. Likewise, Narahari 2017 emphasized the importance of combining Ayurvedic medicine, hygiene practices, and physiotherapy in a structured care model for lymphoedema management [29]. Several studies advocated for integrating NTD-related services into general health systems, rather than maintaining vertical disease-specific programs, to ensure sustainability and accessibility.
2. Diagnostics and therapeutics.
  Another major domain involved the development and deployment of improved diagnostics and treatments across 6 studies(out of 15) [22, 23, 29, 32, 34, 36]. Studies across diseases emphasized the need for point-of-care diagnostics, strain-specific laboratory tests, and affordable therapeutic options. Neevel 2018 and Khazai 2019 stressed the necessity of affordable alternatives to rabies immunoglobulin (RIG) and novel leprosy vaccines [23, 32]. Similarly, Delea 2024 focused on Guinea worm eradication by calling for enhanced diagnostic tools and genomic surveillance technologies [34]. In the case of skin-NTDs, Simpson 2023 proposed the development of diagnostics that support mapping, early detection, and integration of care for overlapping conditions like scabies and Buruli ulcer [36]. Across the board, studies underscored the need for validating new diagnostic models and assessing the feasibility and cost-effectiveness of novel treatments.
3. Surveillance and data systems.
  Six studies (out of 15) stressed the need for integrated surveillance systems that can capture NTD-specific indicators while aligning with national health information systems [27, 28, 30, 31, 33, 34]. This included the establishment of disease registries, tools for real-time data sharing, and feedback loops to inform program adaptation. Studies on vector-borne and zoonotic NTDs pointed to a lack of coordinated epidemiological data on disease burden and transmission [31, 33]. Recommendations included geo-spatial mapping, seroprevalence studies, and multi-sectoral data integration. Enhanced surveillance was deemed crucial for outbreak detection, tracking program performance, and optimizing resource allocation.
4. Community engagement and health education.
  Seven studies (out of 15) acknowledged the critical role of community participation in shaping effective NTD interventions [24–26, 29, 32, 35, 36]. Research priorities in this domain revolved around awareness campaigns, behavioural interventions, and participatory research approaches. Baghotia 2021 and Stienstra 2023 emphasized leveraging the voices of people affected by NTDs (such as PALs for leprosy or community health volunteers in snakebite response) to improve uptake of services, reduce stigma, and enhance early diagnosis [26, 35]. In particular, Aglanu 2023 and Waite 2017 identified the need for community-level sensitization and school-based interventions targeting preventive behaviours and surveillance support [24, 25]. Studies also explored barriers to care-seeking, especially among marginalized populations, and recommended co-creating strategies with communities to overcome these challenges.
5. Integration and coordination across diseases and sectors.
  An emerging domain across several recent studies was the call for integration—of diseases, programs, and sectors [24, 27, 34, 36]. Simpson 2023 proposed integrating mass drug administration (MDA) and morbidity management and disability prevention (MMDP) for multiple skin-NTDs within a single strategy, supported by operational research [36]. Similarly, Waite 2017 emphasized joint indicator development for the WASH–NTD interface, promoting collaboration between sanitation, water, and disease control stakeholders [24]. Delea 2024 proposed a One Health approach for Guinea worm research and eradication, linking human, animal, and environmental health data [34]. Integration was seen as essential not only to reduce duplication of effort but also to ensure that resource-limited health systems can implement NTD programs effectively without parallel structures.
6. Equity, policy, and implementation research.
  The importance of research to support policy development, resource allocation, and context-sensitive implementation models were highlighted in ten out of the 15 included studies [24–28, 30, 32, 33, 35, 36]. The Khazai 2019 study evaluated whether the priorities of the Leprosy Research Initiative were still relevant, emphasizing the need for policy translation and regular consultation with stakeholders [32]. Stienstra 2023 raised concerns about unequal distribution of research capacity across African regions and advocated for regional research hubs to address evidence gaps and build local capacity [35]. Other studies such as Cediel 2013 and Dagenais 2018 focused on how to make prioritization exercises more inclusive, transparent, and responsive to regional health disparities [27, 33]. Issues of stigma, gender equity, and mental health were also featured prominently, particularly in relation to diseases like leprosy and LF. Research was recommended to understand and mitigate the social determinants of NTD burden and to develop multisectoral responses that effectively address these challenges.

## Discussion

This systematic mapping review synthesizes the methodologies, stakeholder engagement strategies, and thematic areas reported in 15 research priority-setting exercises conducted across diverse NTD contexts. While individual exercises have been previously reported, this is, to our knowledge, the first comprehensive synthesis focused exclusively on RPS in NTDs. Our findings reveal significant heterogeneity in how research priorities are identified, the extent of stakeholder inclusion, and the methodological rigor underpinning these processes.

Most included RPS studies employed descriptive or mixed-methods designs and were concentrated in high disease-burden regions, including India, Sub-Saharan Africa, and Latin America. Yet, despite the local relevance, a majority of corresponding authors were affiliated with institutions in high-income countries. This geographic imbalance reflects broader concerns around epistemic inequity in global health research, where LMIC perspectives—particularly those of affected communities—remain underrepresented in agenda-setting processes [37–39]. Global funders allocate an estimated 75% of direct and 70% of indirect NTD funding to leading institutes in nonendemic nations [40]. While such investments modernize laboratory and data infrastructure in HICs, they often perpetuate colonial legacies through sample collection and data extraction from endemic countries without equivalent capacity strengthening [41–43]. Although we found that community members and people with lived experience were involved in over half the studies, their roles were often less explicitly detailed or systematically integrated compared to researchers and policymakers. For example, Pratt et al. (2021) describe how priority-setting processes often include community members only in limited roles rather than as equal partners [44].

Thematically, the most consistent research priorities spanned across service delivery, capacity building, and access to care. This emphasis on strengthening health systems affirms long-standing critiques of vertical disease-specific programming that often neglects the structural determinants of poor NTD outcomes [6]. Studies called for improved training of frontline workers, context-adapted treatment protocols, and enhanced supply chain logistics—challenges echoed in other sectors of global health but made more urgent in the context of chronic underfunding and infrastructural neglect in NTD-affected areas [45–47].

Priority areas in diagnostics and therapeutics also featured prominently, particularly for diseases like rabies, leprosy, and Guinea worm, where existing tools are either unaffordable or suboptimal [48]. The call for point-of-care diagnostics, vaccine development, and alternatives to costly biologicals like rabies immunoglobulin reflects both scientific urgency and health equity concerns [49]. Similarly, the inclusion of community-based strategies and participatory health education highlights a growing recognition that biomedical innovation alone cannot bridge the gaps in NTD care. Evidence from large-scale Visceral Leishmaniasis elimination programs in India that integrated indoor residual spraying, community engagement, and strengthened screening shows that community ownership and behavioural insight are essential for lasting impact [50–54]. This emphasis on community engagement also resonates with the WHO NTD Roadmap’s call for a change in operating culture toward stronger country ownership, defined in part by clearer stakeholder roles and responsibilities in delivering NTD goals [12].

Our findings align with these goals, especially in highlighting operational research needs for integrated service delivery (e.g., joint mass drug administration and MMDP strategies for skin-NTDs), One Health surveillance, and cross-cutting coordination across water, sanitation, and health sectors. However, relatively few studies explicitly adopted or referenced these policy frameworks, suggesting a missed opportunity to align RPS exercises more closely with evolving global strategies. This gap may reflect both the timing of some studies (pre-Roadmap) and the disjointed nature of research governance in the NTD space.

The prevalence of research priorities across the six domains also has implications for future funding direction. The priorities in health systems and service delivery suggests a funding gap in operational research and capacity building at subnational levels, consistent with previous analyses of NTD program implementation [46, 55]. The priorities in equity and implementation research highlight the need for donors to support social science and policy translation work that links research to real-world delivery [56]. Funding for NTDs declined by 41% between 2018 and 2023, increasing dependence on a small number of donors. Transparent, country-led research priority-setting is therefore essential to guide the efficient allocation of limited resources. The WHO report also stresses integrating NTD research into primary health care, adopting rights-based and gender-responsive approaches, and promoting domestic financing for sustainable, equitable research agendas [13].

Our review also highlights methodological inconsistencies across RPS exercises. While some used systematic frameworks like Delphi or CHNRI, others employed less formal or hybrid approaches. Stakeholder engagement methods ranged from multi-stage consultations to single-mode surveys, with limited documentation of processes for consensus-building or conflict resolution. These disparities limit the reproducibility and policy relevance of many RPS outcomes. Use of standardized reporting guidelines such as REPRISE could help to address these gaps by ensuring consistent documentation of processes, stakeholder roles, consensus methods, and conflict-of-interest reporting in RPS exercises [21]. Notably, a few studies disclosed funding sources or conflicts of interest, which raises concerns about transparency and potential bias—particularly in settings where research priorities may influence downstream investments or funding allocations.

### Strengths and Limitations of Study

This review studied the domains of NTD research priority-setting efforts, providing a coherent view of what has been prioritized, by whom, and how. However, this review has several limitation. First, our search was limited to English-language publications, which may have excluded relevant exercises reported in non-English sources or grey literature. Second, the absence of detailed reporting in many studies constrained our ability to compare stakeholder dynamics, funding influences, or alignment with national priorities. Third, while we identified convergence in thematic domains, we did not assess the extent to which identified priorities were implemented or evaluated; which is an important area for future research. Finally, the exclusion of grey literature and early programmatic RPS exercises may bias the evidence base toward donor-funded academic studies published in peer-reviewed journals. This could have under-represented the national or WHO-led consultations, where priorities were defined through policy processes rather than formal research frameworks.

### Implications for Future Research and Practice

The findings highlight the need for research agendas in NTDs to move beyond disease-specific silos and address systemic barriers that constrain progress. Strengthening health systems, improving access to affordable diagnostics and therapeutics, and embedding equity considerations within implementation research are critical. Future exercises should prioritize inclusive stakeholder engagement, especially of community members and persons with lived experiences, and apply structured, transparent methods for prioritization.

The six domains identified in this review provide a practical framework for policy and program guidance. WHO regional offices could use these domains to align national and regional research roadmaps with measurable targets in the NTD Roadmap 2021–2030. For example, priorities under health systems and service delivery can guide integration of NTD care into essential health packages. Diagnostics and therapeutics priorities can inform pooled investments in point-of-care tools across multiple NTDs. Surveillance and data systems can support digital data harmonization in regional monitoring frameworks. Community engagement and health education can shape social mobilization strategies through participatory models. Integration and coordination can support multi-disease MDA planning. Equity, policy, and implementation research can help countries address gender, stigma, and social determinants in national NTD action plans. These domains together form an adaptable template that WHO and national programs can operationalize during roadmap reviews and strategy updates.

To maximize impact, funders and implementers should support the identification of research priorities and also their translation into funded projects, programmatic innovation, and policy reform. Finally, there is an urgent need to build regional research leadership and infrastructure, particularly in LMICs, to ensure that NTD research is driven by those closest to the burden. Strengthening local capacity in priority-setting and implementation research will be key to closing the persistent gap between global ambition and ground realities in the NTD response.

## Conclusion

Despite variations in methodology and scope, six predefined domains—health systems strengthening, access to diagnostics and treatments, data systems, community engagement, integration, and equity; remained central priorities. Future research priority-setting should be transparent, inclusive, and adapted to local contexts. Strengthening local capacity in priority-setting and implementation research is critical to bridging the gap between global goals and on-ground realities, ensuring progress toward the WHO NTD Roadmap targets. The study also calls for caution against disease prioritization approaches that focus only on high-prevalence NTDs and risk overlooking less common NTDs and the populations affected by them.

## Supplementary Information

Below is the link to the electronic supplementary material.

Supplementary Material 1

Supplementary Material 2

## Data Availability

Template data collection forms, data extracted from included studies, data used for all analyses, analytic code, and other materials used in this review are available from the corresponding author upon reasonable request. No new data were generated for this systematic review.
