# Supplementary material for: Research Priority Setting Exercises in Neglected Tropical Diseases: A Systematic Mapping Review
Source: J Epidemiol Glob Health. 2026 Jul 11;16(1):130. doi: 10.1007/s44197-026-00614-y (PMC13642876; doi:10.1007/s44197-026-00614-y)
Supplement: Supplementary file 1 [file 44197_2026_614_MOESM1_ESM.docx]

| **Concept** | Search string | Hits |
| --- | --- | --- |
| 1 | ("NTDs"[Title/Abstract] OR "Tropical diseases"[Text Word] OR "neglected tropical diseas*"[Title/Abstract] OR "Diseases of poverty"[Text Word] OR "Buruli ulcer"[Title/Abstract] OR "Chagas disease"[Title/Abstract] OR "Dengue"[Title/Abstract] OR "Chikungunya"[Title/Abstract] OR "Dracunculiasis"[Title/Abstract] OR "Guinea-worm disease"[Title/Abstract] OR "Echinococcosis"[Title/Abstract] OR "Foodborne trematodiases"[Title/Abstract] OR "Human African trypanosomiasis"[Title/Abstract] OR "Sleeping sickness"[Title/Abstract] OR "Leishmaniasis"[Title/Abstract] OR "Leprosy"[Title/Abstract] OR "Hansen's disease"[Title/Abstract] OR "Lymphatic filariasis"[Title/Abstract] OR "Elephantiasis"[Title/Abstract] OR "Mycetoma"[Title/Abstract] OR "Onchocerciasis"[Title/Abstract] OR "River blindness"[Title/Abstract] OR "Rabies"[Title/Abstract] OR "Scabies"[Title/Abstract] OR "Schistosomiasis"[Title/Abstract] OR "Bilharzia"[Title/Abstract] OR "soil transmitted helminth*"[Title/Abstract] OR "Ascariasis"[Title/Abstract] OR "Hookworm"[Title/Abstract] OR "Trichuriasis"[Title/Abstract] OR "Snakebite"[Title/Abstract] OR "Taeniasis"[Title/Abstract] OR "Cysticercosis"[Title/Abstract] OR "Trachoma"[Title/Abstract] OR "Yaws"[Title/Abstract]) | 1,98,266 |
| 2 | ("research priorit*"[Title/Abstract] OR "Priority setting"[Title/Abstract] OR "Agenda setting"[Title/Abstract] OR "research agenda*"[Title/Abstract] OR "Resource allocation"[Title/Abstract] OR "strategic plan*"[Title/Abstract] OR "Delphi technique"[MeSH Terms] OR "Delphi"[Title/Abstract] OR "consensus conference*"[Title/Abstract] OR "Nominal group technique"[Title/Abstract] OR "Multi-criteria decision analysis"[Title/Abstract] OR "Stakeholder engagement"[Title/Abstract])) | 76,097 |
| Total | ("NTDs"[Title/Abstract] OR "Tropical diseases"[Text Word] OR "neglected tropical diseas*"[Title/Abstract] OR "Diseases of poverty"[Text Word] OR "Buruli ulcer"[Title/Abstract] OR "Chagas disease"[Title/Abstract] OR "Dengue"[Title/Abstract] OR "Chikungunya"[Title/Abstract] OR "Dracunculiasis"[Title/Abstract] OR "Guinea-worm disease"[Title/Abstract] OR "Echinococcosis"[Title/Abstract] OR "Foodborne trematodiases"[Title/Abstract] OR "Human African trypanosomiasis"[Title/Abstract] OR "Sleeping sickness"[Title/Abstract] OR "Leishmaniasis"[Title/Abstract] OR "Leprosy"[Title/Abstract] OR "Hansen's disease"[Title/Abstract] OR "Lymphatic filariasis"[Title/Abstract] OR "Elephantiasis"[Title/Abstract] OR "Mycetoma"[Title/Abstract] OR "Onchocerciasis"[Title/Abstract] OR "River blindness"[Title/Abstract] OR "Rabies"[Title/Abstract] OR "Scabies"[Title/Abstract] OR "Schistosomiasis"[Title/Abstract] OR "Bilharzia"[Title/Abstract] OR "soil transmitted helminth*"[Title/Abstract] OR "Ascariasis"[Title/Abstract] OR "Hookworm"[Title/Abstract] OR "Trichuriasis"[Title/Abstract] OR "Snakebite"[Title/Abstract] OR "Taeniasis"[Title/Abstract] OR "Cysticercosis"[Title/Abstract] OR "Trachoma"[Title/Abstract] OR "Yaws"[Title/Abstract]) AND ("research priorit*"[Title/Abstract] OR "Priority setting"[Title/Abstract] OR "Agenda setting"[Title/Abstract] OR "research agenda*"[Title/Abstract] OR "Resource allocation"[Title/Abstract] OR "strategic plan*"[Title/Abstract] OR "Delphi technique"[MeSH Terms] OR "Delphi"[Title/Abstract] OR "consensus conference*"[Title/Abstract] OR "Nominal group technique"[Title/Abstract] OR "Multi-criteria decision analysis"[Title/Abstract] OR "Stakeholder engagement"[Title/Abstract]) AND "humans"[MeSH Terms] | 444  Searched on 28^th^ March,2025 |

**Search strategy for PubMed**
